# Plant-RNA in Extracellular Vesicles: The Secret of Cross-Kingdom Communication

**DOI:** 10.3390/membranes12040352

**Published:** 2022-03-23

**Authors:** Ornella Urzì, Roberta Gasparro, Nima Rabienezhad Ganji, Riccardo Alessandro, Stefania Raimondo

**Affiliations:** Dipartimento di Biomedicina, Neuroscienze e Diagnostica Avanzata, Università degli Studi di Palermo, 90133 Palermo, Italy; ornella.urzi@unipa.it (O.U.); roberta.gasparro@unipa.it (R.G.); nima.rabienezhadganji@unipa.it (N.R.G.)

**Keywords:** plant-derived extracellular vesicles, cross-kingdom interaction, RNA, biological properties

## Abstract

The release of extracellular vesicles (EVs) is a common language, used by living organisms from different kingdoms as a means of communication between them. Extracellular vesicles are lipoproteic particles that contain many biomolecules, such as proteins, nucleic acids, and lipids. The primary role of EVs is to convey information to the recipient cells, affecting their function. Plant-derived extracellular vesicles (PDEVs) can be isolated from several plant species, and the study of their biological properties is becoming an essential starting point to study cross-kingdom communication, especially between plants and mammalians. Furthermore, the presence of microRNAs (miRNAs) in PDEVs represents an interesting aspect for understanding how PDEVs can target the mammalian genes involved in pathological conditions such as cancer, inflammation, and oxidative stress. In particular, this review focuses on the history of PDEVs, from their discovery, to purification from various matrices, and on the functional role of PDEV-RNAs in cross-kingdom interactions. It is worth noting that miRNAs packaged in PDEVs can be key modulators of human gene expression, representing potential therapeutic agents.

## 1. Introduction

The constant communication between all living organisms, such as plants, bacteria, and animals, attracts the scientific interest of the biomedical community. In general, lipids, proteins, and nucleic acids can be transferred through extracellular vesicles (EVs), which are shuttles involved in cell-to-cell communication.

Extracellular vesicles are lipoproteic particles released by organisms belonging to different kingdoms, and research in recent years has indicated they are mainly involved in cross-kingdom interaction.

The content of EVs consists of various biomolecules, such as proteins, nucleic acids, sugars, and lipids. The main role of EVs is to transfer information to the receiving cells, thereby influencing their functions [1]. Among EVs, different subtypes can be recognized; the International Society for Extracellular Vesicles periodically publishes new guidelines for EV research (MISEV guidelines), and in the latest recommendation, EV subtypes are distinguished based on their physical properties, such as dimension and density (small EVs or medium/large EVs), the biochemical composition (CD63+/CD81+—EVs, Annexin A5-stained EVs, etc.), and on the cell of origin [1]. The discovery of the presence of both messenger and small noncoding RNAs inside mammalian EVs in 2007 revolutionized the field of cell–cell communication [2]. Many studies correlated the ability of EVs to modulate the functional properties of target cells with their RNA cargo [3,4,5]. RNAs contained in EVs, once internalized, can trigger molecular and phenotypic changes in the recipient cell. EV-mRNAs can be translated into functional proteins, while EV-ncRNAs can engage complex networks of interactions that regulate gene expression [6]. For instance, the EVs released by multiple myeloma cells are enriched in some miRNAs that can affect the expression of genes involved in osteogenic differentiation in mesenchymal stem cells. Among them, miR-129-5p downregulates the expression of SP1, a positive regulator of osteogenic differentiation, and alkaline phosphatase in mesenchymal stem cells; thus, inhibiting osteoblastic differentiation [3]. RNAs delivered by EVs are also involved in pre-metastatic niche formation. Conigliaro et al. demonstrated that EVs released by CD90+ liver cancer cells carry lncRNA H19, which promotes tube formation and the cell–cell interaction of endothelial cells; thus, favoring angiogenesis [7]. EV-RNAs play a key role, not only in pathological, but also in physiological, processes; understanding their mechanisms of action could lead to new therapeutic strategies. Li and colleagues demonstrated the beneficial effects of EVs isolated from M2 microglia cells in mice with ischemic stroke. They found that M2 microglial EVs were enriched in miR-124, which suppresses astrocytes proliferation through the inhibition of the STAT3 pathway; thus, reducing glial scar formation [8].

Nowadays, it is known that extracellular vesicles are also released by plants. Although the guidelines provided in MISEV [1] are also valid for those studying vesicles within the plant kingdom, the growing interest in plant-derived extracellular vesicles (PDEVs) requires a more detailed standardization, recently discussed by Pinedo and colleagues [9]. First, the nomenclature of EVs isolated from plants should be revised. PDEVs in the literature are named using different terms, such as nanovesicles [10], nanoparticles [11], microvesicles [12], exosomes [13], and exosome-like vesicles [14]. However, information on their biogenesis is still lacking, thereby complicating the establishment of a clear nomenclature. Pinedo et al. suggested using the term ‘plant EVs’ only for particles isolated from the apoplastic fluid, while for vesicles obtained from plant tissues after disruptive processes, they recommend the term ‘plant-derived nanovesicles’, since the origin of the obtained vesicles is dubious [9]. In this review, we will use the term ‘PDEVs’ to refer to both EVs from apoplastic fluid and those obtained by destructive methods. In addition to the appropriate nomenclature, isolation methods and the identification of specific markers are critical for PDEVs standardization; these points will be discussed in the subsequent paragraph.

The information contained in this review article aims to summarize the history of the research on plant-derived extracellular vesicles, as well as to report evidence on their role in cross-kingdom interaction. Our manuscript will focus on the PDEV-RNA component. The PubMed database was used as the source for the retrieval of the literature included in this work.

**Figure 1:**
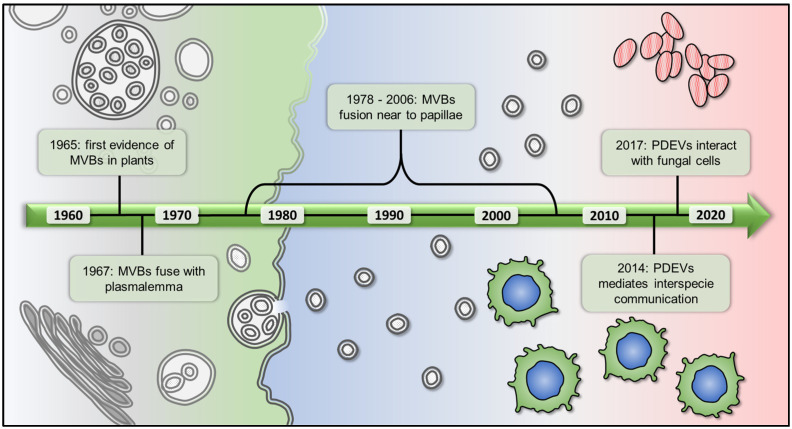
Timeline of plant-derived extracellular vesicles research. The first observation of PDEVs came from electronic transmission microscopy in the 1960s. For several years they were correlated to the response against pathogens, until it their capability to mediate the cross-kingdom communication was demonstrated. MVBs, multivesicular bodies; PDEVs, plant-derived extracellular vesicles.

The figure below (Figure 1) represents a chronological summary of the discovery of the EVs in the plant kingdom that will be discussed in this review.

## 2. The Story of Plant EVs, from Their Discovery to the Purification from Different Matrices

Similarly to other living organisms, plants produce extracellular vesicles (EVs), and the discovery of plant-derived extracellular vesicles can be attributed to Jensen, who in 1965 found multivesicular bodies (MVBs), and, thus, intraluminal vesicles, in cotton [15]. Two years later, Halperin and Jensen analyzed the ultrastructure of clumps in suspension cell cultures of wild carrot and found the presence of numerous MVBs able to fuse with the plasmalemma and release their contents into the wall space [16]. In 1978, Politis and Goodman observed membrane-bound vesicles in the microfibrillar material forming the cell wall apposition of tobacco leaves inoculated with non-pathogenic *Pseudomonas pisi* [17]. Similarly, other studies found that, during pathogen attacks, plant cells have MVBs that are observed in various states of fusion with the plasmalemma. Most of these fusion events occur near the papillae; extracellular defense structures that block pathogen entry into the cell [18,19,20]. PDEVs were isolated from the apoplastic fluid for the first time in 2009 by Regente et al. [21]. The initial studies suggested that EVs in plants contributed to the development of early defense structures, in response to pathogens; however, their biological functions remained obscure for a long time. Some years later, it was found that PDEVs can mediate interspecies communication [22,23,24], for instance after oral administration grapes and grapefruit-derived EVs were taken up by intestinal macrophages in mice and exerted anti-inflammatory and antioxidant effects [22]. Cross-kingdom interaction also includes fungi; in 2017, Regente and colleagues demonstrated that PDEVs isolated from sunflower can interact with fungal cells, inhibiting fungal growth and causing cell death [25]. This observation was supported by the study of Rutter and Innes, who demonstrated that PDEVs secretion was enhanced in infected *Arabidopsis thaliana* leaves, contributing to innate immunity [26].

**Figure 2:**
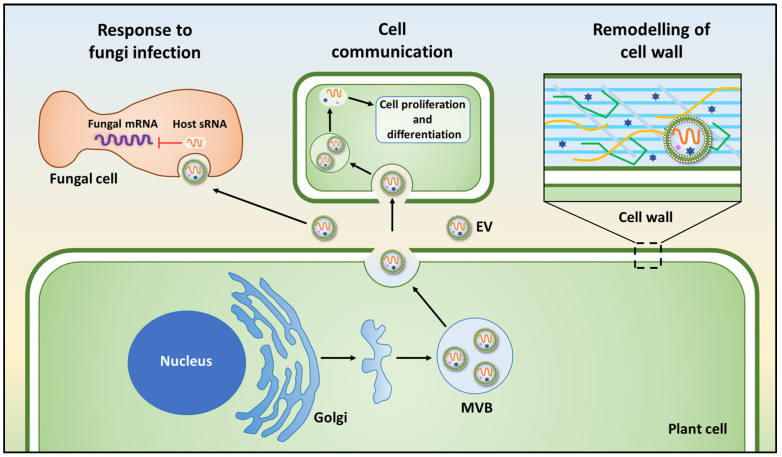
The physiological roles of plant-derived extracellular vesicles. The main functions of PDEVs are (i) response to fungi infection; (ii) cell–cell communication; (iii) cell wall remodeling. EV, extracellular vesicle; MVB, multivesicular body; sRNA, short RNA, mRNA, messenger RNA.

In the figure below, we have summarized the results of the first studies on the functional role of PDEVs (Figure 2).

The interest of the scientific community in PDEVs increased when the presence of microRNAs able to bind target genes in human cells was demonstrated [27]. Moreover, recently the mechanism of small RNA sorting into PDEVs was partially elucidated by He et al., who described the involvement of RNA binding proteins, such as Argonaute 1, RNA helicases, and Annexins, in this process [28]. We will analyze the functional role of PDEV-RNA in the following paragraph.

**Figure 3:**
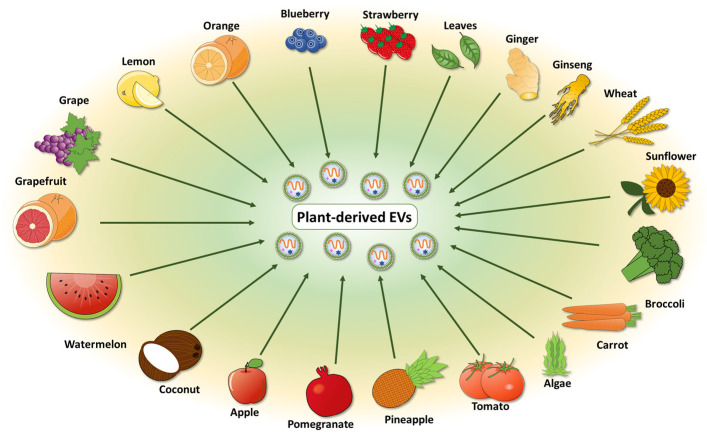
The sources of plant-derived extracellular vesicles. PDEVs can be isolated from various edible plants, such as fruits and vegetables. The starting matrix can be represented by tissues or organs, juice, leaves, seeds, and roots.

As mentioned previously, one of the main challenges in the field of PDEVs remains the standardization of the isolation method, and consequently the nature of isolated EVs [9]. PDEVs have been isolated from the tissues, organs, apoplastic fluid, and juice of several plant species, such as lemon [10,29,30,31,32,33], grapefruit [22,27,30,31,34,35,36,37,38], tomato [27,39,40,41,42], and Arabidopsis [26,28,43,44,45,46,47,48] (Figure 3). Only a minority of studies have purified EVs from the extracellular apoplastic fluid [21,25,26]; most of the works were conducted using other methods to isolate PDEVs, which mainly consisted of organ and tissues disruption. The first technique ensures the recovery of most EVs, while the second method causes the contamination of the sample with intracellular compartment and artificial nanoparticles [9].

Although new techniques for PDEVs isolation have emerged in the last year, such as immunocapture purification [28] and the aqueous two-phase system [49], differential centrifugation remains the most widely used method [40,46,50]. Some groups adopted isolation protocols similar to those used for mammalian EVs. Briefly, the starting material is squeezed, and the obtained juice is subjected to multiple steps of centrifugation: low-speed centrifugation (500–3000× *g* for about 10 min), intermediate speed centrifugation (2000–10,000× *g* for about 30 min), and ultracentrifugation (100,000–150,000× *g* for 1.5–2 h) to obtain a PDEV pellet. However, since ultracentrifugation also sediments other vesicles, proteins, and protein/RNA aggregates; while, some protocols add a subsequent density gradient ultracentrifugation using iodixanol or sucrose/deuterium oxide to separate PDEVs from contaminants [50,51,52]. On the other hand, Regente and other researchers have found that PDEVs can pellet at a lower speed, such as 40,000× *g* [21,26,53].

Once isolated, PDEVs should be appropriately characterized using various methods, including nanoparticle tracking, flow-cytometry, transmission electron microscopy, and other forms of electron microscopy. The PDEV cargo is complex and heterogeneous, for this reason, the best methods for studying their content are based on omics analysis. In the last years, several studies were carried out to characterize the proteomic profile of EVs isolated from different plant species [10,21,26,30,42,54]; these findings could help to identify possible PDEVs markers. Among the proteins identified in PDEVs, the most represented are heat shock protein 70 (HSP70), S-adenosyl-homocysteinase, and glyceraldehyde 3 phosphate dehydrogenase, which were found in EVs isolated from olive pollen [55], *Nicotiana benthamiana* [56], *Arabidopsis thaliana* [26], and sunflower [25]; these proteins could be candidates as PDEVs markers, even if further studies are needed to confirm their presence in EVs isolated from the majority of plants. Other protein families well represented in PDEVs are that of aquaporins, present in citrus- [10,30,52] and grape-derived EVs [56]; and annexins, found in citrus- [10,30], sunflower- [21], and Arabidopsis-derived EVs [26]. Besides proteins, the lipid characterization of PDEVs has attracted the interest of the scientific community, since this component seems to be strictly correlated to their biological functions [23]. The main lipid species identified in PDEVs are phosphatidic acid (PA), found in EVs from sunflower [21], grape [56], ginger [23], *Uvae-ursi folium*, *Craterostigma* *plantagineum*, and *Zingiberis rhizome* [57]; phosphatidylethanolamine (PE), described in EVs-derived from grapefruit [58], grape [56], *Craterostigma plantagineum*, and *Zingiberis rhizome* [57]; and phosphatidylcholine (PC), present in grapefruit- [58] and *Craterostigma plantagineum*-EVs [57]. Moreover, PDEV cargo includes several metabolites, such as sulforaphane [59], shogaol [60], and flavonoids [36,56,58], which could explain the PDEV-mediated beneficial roles in plant–mammalian interactions. On the other hand, a recent study published by Stanly and colleagues showed that both micro-and nano-vesicles isolated from strawberry carry functional allergens, including Fra a 1, Fra a 3, and Fra a 4 [51]; thus, demonstrating for the first time that PDEVs can also transport this type of molecule.

PDEVs also contain different species of RNA; this topic and the correlated studies will be discussed in the following paragraphs.

## 3. Functional Role of PDEV-RNAs in Cross-Kingdom Interactions

**Figure 4:**
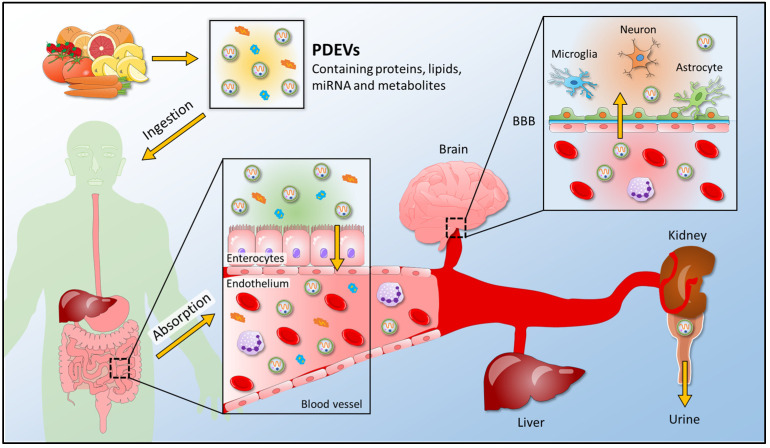
A schematic view for the uptake of plant-derived extracellular vesicles into the human body. PDEVs containing proteins, lipids, miRNA, and metabolites enter the human body after edible plant ingestion. In the gastrointestinal tract, where food is digested, PDEVs are absorbed and enter the bloodstream; thus, reaching the final recipient organs, such as the brain, liver, and kidney. PDEVs release their content in target organs and exert their biological properties. They can cross the BBB and reach the cells of the central nervous system, or they can be found in the urine of plant-eating humans. PDEVs, plant-derived extracellular vesicles; BBB, blood-brain barrier.

Discoveries correlated to plant-derived extracellular vesicles are becoming an essential starting point to study cross-kingdom communication; as a consequence, several studies have been carried out to analyze the interaction between plant vesicles and mammalian targets (Figure 4).

### 3.1. Functional Roles of Plant Extracellular Vesicles in Plant-Mammalian Communication

Plant-derived extracellular vesicles are also known for their specific proprieties, having a low immunological risk, and above all for their higher bioavailability. In terms of safety, plants do not have any zoonotic or human pathogens. Higher bioavailability is one of their most interesting features in cross-kingdom interaction: components packed into extracellular vesicles, including miRNAs, are protected by the lipid layer, in order to avoid their degradation. Consequently, cross-kingdom interaction paves the way for new applications of plant-derived extracellular vesicles and other findings on the regulation of mammalian targets as mediated by plants’ miRNAs. PDEVs are also known for their anti-inflammatory and anti-cancer properties. For example, EVs derived from grape [56], grapefruit [58], ginger [61], ginseng [62], and mulberry bark [63] have been reported for their effects in treating colitis, inflammation, and cancer. Ginseng is also used to enhance neurogenesis: Xu et al. reported the efficacy of extracellular vesicles derived from ginseng in transferring active nucleic acids to stem cells. In particular, miRNAs packed into EVs derived from ginseng can induce the neural differentiation of BMSCs. Ginseng-EVs upregulate PI3K signaling and induce the activation of neural differentiation in vitro. In vivo, ginseng-EVs promote neural restoration through upregulating PI3K signaling, increase nerve regeneration by promoting neurotrophin expression, and influence the Ras/Erk pathways. In addition, it was reported that mtr-miR-159a, a miRNA packed into ginger-EVs, can upregulate the PI3K signaling pathway [13]. Furthermore, Sundaram et al. recently reported that oral administration of garlic-EVs resolves brain inflammation and obesity in mice under a high-fat diet. These EVs are taken up by microglial cells, and inhibit brain inflammation via IDO1-mediated AHR pathway and c-Myc-mediated c-GAS/STING inflammatory pathway [64]. Cao et al. demonstrated, for the first time, that EVs isolated from the roots of *Panax ginseng* can induce M1-like macrophage polarization via the Toll-like receptor 4/myeloid differentiation antigen 88 signaling pathway and enhance the production of total reactive oxygen species (ROS), to induce apoptosis of mouse melanoma cells. Consequently, it was found that ginseng-derived EVs can inhibit tumor growth in mice [62]. Furthermore, another interesting study reported that leaf-derived extracellular vesicles from *Dendropanax morbifera* can inhibit tyrosinase activity and also reduce melanin content in melanoma cells, representing a candidate for the development of new anti-melanogenic agents [65]. Kim et al. demonstrated in 2020 that *Dendropanax Morbifera*-EVs exerts cytotoxic effects on malignant breast and skin tumor cells, without affecting normal cells, while *Pinus densiflora*-EVs are cytotoxic toward malignant skin tumor cells, but not toward normal cells. These results indicate that EVs derived from plant sap are selectively cytotoxic against tumor cells [66]. Recently, it was found that EVs isolated from apple possess anti-inflammatory properties, since they can downregulate the expression of pro-inflammatory cytokines, such as IL-8 and IL-1β, in human macrophages. The anti-inflammatory activities of apple-EVs could be explained by their ability to upregulate some miRNAs in target cells, such as miR-146a-5p, which is involved in NF-κB (Nuclear Factor kappa B) regulation [67].

Interestingly, Zhang [68] and others [69,70] highlighted for the first time that the microRNAs (miRNAs) present in plant extracts, introduced by diet, can control gene expression in human cells. Indeed, miRNAs have emerged as novel signaling molecules, to mediate intercellular communication. MiRNAs are small ∼21–22 nt non-coding RNAs, which are known to be regulators of essential biological processes in animals and plants. In 2012, it was found that food-derived miRNAs can provide cross-kingdom regulation. In particular, plant-derived miRNA can regulate gene expression with complementarity to their target mRNA, even if the mammalian mRNAs only possess partial homology to the targets [71]. Despite this, several studies focus on the role of the exogenous plant’s miRNAs in the gene expression of mammalian cells regulation. Interesting findings regarding plants’ extracellular vesicles concern the presence of small RNAs, especially miRNAs, which can represent a new class of cross-kingdom modulators. Recent evidence demonstrated that plants’ miRNAs may be considered a new class of micronutrients responsible for the medical properties of plants. Generally, plant miRNAs are involved in gene targeting, and the literature reports their beneficial role in some pathological conditions such as cancer. For example, plant miR-159 is correlated with a decrease in breast cancer incidence and progression. In particular, TCF7 is a mammalian target for plant miR-159, and it has shown the anti-proliferative function of miR-159 in breast cancer cells; demonstrating that a plant miRNA can influence cancer cell growth. Furthermore, a synthetic mimic of miR-159 can inhibit proliferation by targeting the TCF7 that encodes a Wnt signaling transcription factor, leading to a decrease in MYC protein levels [72]. Moreover, miR-58 derived from salvia has an effect on the reduction of Akt/mTOR (mammalian target of rapamycin) signaling, also facilitating autophagy [73]. It was also recently reported that miR-167e-5p represses intestinal cell proliferation by targeting β-catenin, and that the natural oeu-sRNAs decrease the protein expression of miR-34a mRNA targets, reducing proliferation and increasing apoptosis in different tumor cells [74]. However, in other studies, the intake of plant miRNAs through the diet has been challenged [75,76,77]. Snow et al. selected three highly expressed plant miRNAs (miR-156a, miR-159a, and miR-169a) and analyzed their levels in the plasma of healthy subjects after the intake of fruits containing these miRNAs. However, the plant miRNAs were undetectable in the plasma of healthy subjects. Similar data were obtained in mice: the authors fed mice with a vegetarian diet, but they did not detect any significant increase in plant miRNAs expression, both in the plasma and organs of mice [75]. These observations were supported by another study in which pigtailed macaques were fed with a miRNA-rich plant-based diet and then plant miRNAs were analyzed in animal blood through RT-qPCR, after 1, 4, and 12 h. It was found that the amplification of plant miRNAs was variable and possibly non-specific; thus, refuting the hypothesis of horizontal transfer of small RNAs from plant to mammals through oral administration; even if the study was carried out with only two macaque subjects [76]. Moreover, another group failed in the detection of plant miRNAs in mice fed with a rice-based diet for 1, 3, and 7 days. They measured the levels of rice miRNAs through miRNA sequencing and qPCR in plasma and liver of mice fed with rice-based diet, but there were no differences compared to the control group (mice fed with synthetic chow) [77]. Nevertheless, the work of Zhang was further accompanied by others that support the idea that plant miRNAs can be found in the blood, urine, and tissues of plant-eating animals [78,79]. It could be possible that some studies failed to detect plant miRNAs in animal tissues due to small dosages and a short time of exposure to the fed RNA [80].

Emerging studies have pointed out the role of miRNAs, packed in PDEVs, in the regulation of mammalian genes. Potestà et al. demonstrated that EVs derived from *Moringa oleifera* seeds, which carry plant miRNAs, can naturally penetrate inside human tumor cells and have proapoptotic effects. In particular, these EVs can modulate activities related to the viability and apoptosis in tumor cell lines, thanks to the regulation being mediated by miRNAs of BCL2 protein, which is one of the main factors that affects tumorigenesis in cell lines of epithelial origin [81]. Another interesting study demonstrated that bitter melon-derived extracellular vesicles can induce the apoptosis of oral squamous cell carcinoma (OSCC) cells. In particular, *NLRP3*, which is a component of the innate immune system, is able to promote tumor growth and metastasis in OSCC [82] and can be downregulated by miRNA-22, promoting cancer cell growth repression [83]. This is considered an interesting key point in cross-kingdom interaction because, as was said before, plant miRNAs can reduce the expression of target genes in animals and contribute to the function of tissues. Therefore, Yang et al. detected twenty-four kinds of miRNAs packed into EVs derived from bitter melon, and 11 of them had the potential to regulate the expression of NLRP3 mRNA, downregulating the protein expression of *NLRP3*. Furthermore, EVs derived from bitter melon combined with 5-fluorouracil enhanced OSCC apoptosis via augmented ROS generation; thus, increasing the cytotoxic effects and reducing the 5-fluorouracil drug resistance [84].

In addition to PDEVs’ anti-cancer effect, other biological and functional properties known in the literature are their anti-inflammatory and anti-oxidant role in human and mice models. It has been shown that PDEVs are taken up by macrophages and exhibit anti-inflammatory effects; for example, ginger-derived EVs are preferentially taken up by intestinal macrophages or monocytes, and, therefore, induce anti-inflammatory mediators. In one of these studies, colitis was induced in mice using dextran sulfate sodium, and the mice were subsequently treated with ginger-derived EVs. The results indicated that these EVs exhibited anti-inflammatory effects, with a decrease in lipocalin-2, a known biomarker for intestinal inflammation [61]. It was also found that mice treated with ginger EVs showed significant downregulation of pro-inflammatory cytokines, such as IL-6 and TNF-α, as well as the upregulation of the anti-inflammatory cytokine IL-10. Moreover, it was demonstrated that EVs isolated by *Citrus sinensis* can penetrate intestinal epithelial cells and positively modulate the expression of anti-inflammatory genes and tight junction. In particular, *Citrus sinensis*-EVs can modulate the expression of important genes related to inflammatory pathways, such as *HMOX-1* and *ICAM1*, or the restoration of intestinal permeability related to *claudins* and *occludin* [85]. In addition, plant EVs resist gastric pepsin solution and intestinal pancreatic and bile extract solutions, indicating their potential for influencing cell biology through ingested foods [58] and suggesting the possible use of food EVs as safe therapeutic agents.

MiRNAs’ role in the anti-inflammatory effect of PDEVs has attracted many researchers: several studies were carried out to find the correlation between miRNAs packed into plant-derived extracellular vesicles and inflammatory response targets. For example, an interesting finding reports that mammalian genes involved in the regulation of inflammatory cytokines (IL-5 and IL-6) can be targeted by PDEV miRNAs, suggesting that these plant vesicle-derived miRNAs can potentially regulate mammalian mRNAs and biological pathways. miRNAs can, indeed, directly target genes encoding inflammatory factors. For example, miR-5781 in soybean-EVs can directly target *IL-17A*. MiR-4995 packed into tomato-EVs can target *IL-5*, while miR-1078 in ginger-EVs can target *IL-6* [27]. Consequentially, it is important to consider the potential roles that these PDEV-derived miRNAs can play in host health and disease, paving the way for new cross-kingdom interaction findings [86]. Interestingly, a therapeutic effect of plant miRNAs in the prevention of chronic inflammation was shown in a mouse model of human multiple sclerosis. In particular, the impact of plant miRNAs on dendritic cells was explored, a component of the innate immune system in the gut and responsible for instructing T cells. *Fragaria vesca* miR-168 can reduce the inflammation mediated by TLR agonists via a TLR3-mediated mechanism: treatment with plant miRNA can reduce inflammation and prevent symptoms of multiple sclerosis in mouse models. In particular, it was demonstrated that miR-168 can reduce the inflammatory response, and this effect was associated with a decreased expression of TRIF transcript, an essential adaptor protein required for innate immune responses mediated by TLR3 [87]. Moreover, Link et al. found detectable levels of plant miR-168 in human feces, normal gastric, and colon cancer mucosa [88], suggesting potential interspecies activity.

Another role mediated by PDEVs is their antioxidant effect on human cell models. An interesting study reported the antioxidant effect of strawberry-derived extracellular vesicles. These EVs were isolated from the strawberry juice of *Fragaria x ananassa* and they could prevent oxidative stress in human mesenchymal stromal cells in a dose-dependent manner. The analysis of their cargo revealed the presence of small RNAs and miRNAs; in particular, a specific enrichment of miR-166g was found [89]. The antioxidant role of PDEVs has also been demonstrated, thanks to the analysis of extracellular vesicles derived from other plant species. For example, carrot-derived EVs were investigated for their antioxidative and apoptotic effects in cardiomyoblasts and neuroblastoma cells. Carrot-derived EVs can inhibit the ROS generation induced by H_2_O_2_ and apoptosis induction. In particular, Kim et al. reported that carrot-derived EVs can inhibit the decrease in *Nrf-2* expression in cardiomyoblast cells, thereby protecting cells from oxidative stress. In contrast, the decrease in *HO-1* expression is reduced when cells are supplemented with carrot-derived EVs. Similar expression patterns are observed for *NQO-1* expression, indicating that carrot-derived EVs effectively inhibit the decrease in the expression of this antioxidative protein [90].

### 3.2. Plant Extracellular Vesicles in Plant-Microbe Interaction

Plants and animals can be under constant pathogen attack. Some pathogens and pests deliver small RNAs (sRNAs) into host cells to suppress host immunity. Conversely, hosts also transfer sRNAs into pathogens and pests to inhibit their virulence. Emerging findings have revealed that some sRNAs can, indeed, travel between hosts and interact with microbes and fungi to silence target genes. In particular, RNA interference (RNAi) is one of the primary adaptive defense mechanisms that can regulate plant immune responses against several kinds of pathogens. For example, plants such as cotton can export plant-specific miRNAs into their fungal pathogens to induce cross-kingdom gene silencing and confer disease resistance. Cotton plants produce miR-166 and miR-159, which are exported to the hyphae of pathogenic fungus *Verticillium dahlia*. These miRNAs target a Ca^2+^—dependent cysteine protease and an isotrichodermin C-15 hydroxylase that are crucial for virulence [91].

Intriguingly, PDEV secretion is increased by pathogen infection, suggesting that EVs play important roles in plant immunity [26]. In addition to mammalians, plant-derived EVs can, indeed, mediate the communication with pathogens through RNAi. In particular, emerging studies have shown that sRNA derived from plants can silence microorganism and fungi target genes [92]. As a consequence, the discovery of EV-mediated trafficking of sRNAs from plant hosts to fungal pathogens has given rise to many exciting questions. For instance, Cai et al. discovered that small interfering RNAs are delivered by Arabidopsis into *B. cinerea* cells, promoting the silencing of fungal genes [43]. Further studies have revealed that cross-kingdom RNA trafficking, from the host into the pathogen, to induce the silencing of pathogenic genes, depends on EVs [93,94]. Arabidopsis sRNAs transported into *B. cinerea* cells are also packed into plant-derived EVs, indicating that EV-mediated transport is one of the most relevant pathways for the cross-kingdom trafficking of sRNA. Cai et al. also demonstrated that sRNA-containing vesicles accumulate at the infection sites and are taken up by the fungal cells. Moreover, transferred host sRNAs induce silencing of fungal genes critical for pathogenicity. Consequently, it was reported that plant extracellular vesicles play an essential role in cross-kingdom sRNA trafficking between Arabidopsis and the fungal pathogen *B. cinerea*, because Arabidopsis secretes EVs to deliver host sRNAs into fungal cells to silence virulence-related genes [43].

In addition, it is known that plant cells can release EVs containing transport RNAs, defense compounds, and signaling lipids, suggesting that plant EVs can also function as important mediators in plant–microbe interactions [26,95]. Kalarikkal et al., using an in silico approach, showed that a high abundance of miRNAs in PDEVs can target SARS-CoV-2 genes [35]. In particular, target prediction analysis was carried out using RNA hybrid software with stringent prediction criteria. Critical parameters were set during target prediction to identify SARS-CoV-2 targeting miRNAs, as reported by the authors. To confirm the specificity of miRNA binding to SARS-CoV-2 genome, the authors aligned the miRNA binding site sequence within the SARS-CoV-2 genome with global isolates of SARS-CoV-2 and SARS-CoV sequence, using MEGA software. They showed that gma-miR-4995 and mdm-miR-1511, which are respectively packed into coconut- and pear-derived EVs, can target *SPIKE*, the gene involved in encoding the structural protein spike. Moreover, they found that Osa-miR-530-5p, a miRNA carried by PDEVs, can indirectly inhibit the synthesis of *ORF1b*, by not allowing ribosomal slippage, and, thus, preventing the replication of SARS-CoV-2 [35]. In the same study, the authors validated the relative expression of six miRNAs and their differential enrichment; these include (i) miRNAs that are expressed equally in both ginger and grapefruit PDEVs (gma-miR-166 m and mtr-miR-156a), (ii) miRNAs that show higher enrichment in grapefruit PDEVs (bdi-miR-5059 and osa-miR-5077), and (iii) miRNAs that show higher enrichment in ginger PDEVs (aqc-miR-159 and gma-miR-6300). In addition, using an in silico approach, the authors identified other PDEV-derived miRNAs targeting the SARS-CoV-2 genome; gma-miR-6300 target ORF3a, which codifies for an accessory protein that inhibits autophagy by blocking the fusion of autophagosomes with lysosomes [96]. In addition, aqc-miR-159 (derived from ginger PDEVs) and zma-miR-164b-3p (derived from pear PDEVs) can respectively target SARS-CoV-2′s M and N genes, codifying membrane glycoprotein and nucleocapsid protein. Finally, pvu-miR-482-5p, a miRNA packed into pea’s PDEVs, targets ORF8, another crucial gene involved in the immune evasion of SARS-CoV-2 [97]. However, as is underlined by the authors, these results are based on in silico analysis; therefore, further in vitro and in vivo investigations are necessary to evaluate the anti-viral capacity of the microRNAs carried by PDEVs. Evidence resulting from future studies may be critical for the development of an efficient therapy for COVID-19.

Interestingly, another study demonstrated that PDEV-derived miRNAs inhibit lung inflammation, induced by exosomal SARS-CoV-2 Nsp12; Nsp12 is delivered by lung epithelial cell exosomes to macrophages, leading to the activation of the macrophages via NF-κB. Furthermore, a large number of ginger-EV miRNAs can potentially bind to multiple sites of the SARS-CoV-2 viral genome. This study demonstrated that ginger-derived EVs contain aly-miR396a-5p, which can inhibit NF-κB-mediated inflammation and apoptosis in the lungs of mice. These results pave the way for the possible application of PDEV-based therapy, since the activation of NF-κB-mediated pathways plays an essential role in many inflammatory diseases, including COVID-19 [14].

In the following table (Table 1), we have summarized the studies discussed above, focusing on the PDEV sources and miRNA functional roles.

## 4. Conclusions

In conclusion, the role that extracellular vesicles of plant origin may play in cross-kingdom communication has attracted the attention of the scientific community, due to their ability to regulate human targets. Above all, miRNAs packaged in PDEVs may be key modulators in the regulation of human genes, representing a possible strategy in human therapies. In addition, their higher bioavailability may encourage the application of PDEVs in some pathological conditions, such as cancer, inflammation, and response to oxidative stress. Despite this, more studies need to be carried out to validate the beneficial effects of PDEVs and, most of all, to understand the mechanism behind human target regulation mediated by EV-miRNAs.

## Figures and Tables

**Table 1:** Origin and functional role of microRNAs packed into different plant-derived extracellular vesicles.

PDEV Origin	microRNA	Functional Role	References
Bitter melon	miR-156 d, miR-162, miR-166 5p, miR-167, miR-172,miR-390, miR-394, miR-396 3p, miR-399, miR-529, miR-2111 5p	Potential role in the regulation of NLRP3 mRNA	[84]
Soybean	miR-5781, miR-4996, miR-5671a	Regulatation of interleukin 17A, interleukin 10, interleukin 33	[27]
	gma-miR-6300	Targeting gene ORF3a of SARS-CoV-2	[35]
	mtr-miR-156a	Targeting gene ORF1ab of SARS-CoV-2	
Hami melon	miR-164a	Regulatation of interleukin 16	[27]
	ath-miR-164b-5p, zma-miR-398b-5p, cme-miR-530b, cme-miR-399d	Targeting gene ORF1ab of SARS-CoV-2	[35]
Orange	miR-398b	Regulatation of interleukin 1, alpha	[27]
Ginger	miR-1078	Regulatation of interleukin 6	
	miR-7267-3p	Suppression of *Lactobacillus rhamnosus* monooxygenase ycnE mRNA, in the gut microbiome	[23]
	aly-miR396a-5p	Inhibition of the expression of inflammatory cytokines induced by Nsp12 of SARS-CoV-2; suppression of the SARS-CoV-2 cytopathic effect by inhibiting the expression of the viral S and Nsp12	[14]
	rlcv-miR-rL1-28-3p	Suppression of the SARS-CoV-2 cytopathic effect by inhibiting the expression of the viral S and Nsp12	
	gma-miR-6300	Targeting gene ORF3a of SARS-CoV-2	[35]
	aqc-miR-159	Targeting gene M of SARS-CoV-2	
Tomato	miR-4995	Regulatation of interleukin 5	[27]
	gma-miR-6300	Targeting gene ORF3a of SARS-CoV-2	[35]
	gma-miR-4375, zma-miR-398b-5p, bdi-miR-5059, osa-miR-5077	Targeting gene ORF1ab of SARS-CoV-2	
	sly-miR-1919a	Targeting gene ORF10 of SARS-CoV-2	
Fragaria	miR-166g	Disruption of the morphogenesis of leaves	[89]
Moringa oleifera	*mol*-miR160h, *mol*-mir482b, *mol*-mir166, *mol*-mir 159c, *mol*-mir2118a, *mol*-mir167f-3p, *mol*-mir156e, *mol*-mir395d, *mol*-mir393a, *mol*-mir397a, *mol*-mir858b, *mol*-mir396a	Potential regulation of proapoptotic and antiapoptotic targets	[81]
Walnuts	miR-156c, miR-159a	Regulation of mammalian TNF-α signaling pathway in adipocytes and regulate inflammation	[69]
Coconut	gma-miR-4995	Targeting gene SPIKE of SARS-CoV-2	[35]
	mtr-miR-156a	Targeting gene ORF1ab of SARS-CoV-2	
Pear	mdm-miR-1511	Targeting gene SPIKE of SARS-CoV-2	
	zma-miR-164b-3p	Targeting gene N of SARS-CoV-2	
Pea	pvu-miR-482-5p	Targeting gene ORF8 of SARS-CoV-2	
	gma-miR-156f	Targeting gene ORF1ab of SARS-CoV-2	
Blueberry	zma-miR-398b-5p		
Grapefruit	bdi-miR-5059, osa-miR-5077		
Kiwifruit	osa-miR-530-5p		
Grapes	vvi-miR-3630, vvi-miR-156a/n, vvi-miR-169r/u		

## Data Availability

Not applicable.
